# Surfactant Lipidomics of Alveolar Lavage Fluid in Mice Based on Ultra-High-Performance Liquid Chromatography Coupled to Hybrid Quadrupole-Exactive Orbitrap Mass Spectrometry

**DOI:** 10.3390/metabo9040080

**Published:** 2019-04-25

**Authors:** Rui Yang, Ying Zhang, Wenjuan Qian, Linxiu Peng, Lili Lin, Jia Xu, Tong Xie, Jianjian Ji, Xiuqin Zhan, Jinjun Shan

**Affiliations:** 1 Jiangsu Key Laboratory of Pediatric Respiratory Disease, School of Medicine and Life Sciences, Nanjing University of Chinese Medicine, Nanjing 210023, China; 20171349@njucm.edu.cn (R.Y.); 20161325@njucm.edu.cn (W.Q.); 2 Medical Metabolomics Center, Nanjing University of Chinese Medicine, Nanjing 210023, China; penglx01@163.com (L.P.); linnj@njucm.edu.cn (L.L.); njxj0106@126.com (J.X.); sunnyxyl1021@163.com (T.X.); 3 Institute of Pediatrics, Nanjing University of Chinese Medicine, Nanjing 210023, China; Jijj@njucm.edu.cn; 4 Genome Center of UC Davis, NIH West Coast Metabolomics Center, Davis, CA 95616, USA; yzhang1088@gmail.com

**Keywords:** surfactant lipidomics, high-resolution mass spectrometry, acute lung injury, bronchoalveolar lavage fluid, lipopolysaccharide

## Abstract

Surfactant lipid metabolism is closely related to pulmonary diseases. Lipid metabolism disorder can cause lung diseases, vice versa. With this rationale, a useful method was established in this study to determine the lipidome in bronchoalveolar lavage fluid (BALF) of mice. The lipid components in BALF were extracted by liquid–liquid extraction (methanol and methyl tert-butyl ether, and water). Ultra-high-performance liquid chromatography coupled to hybrid Quadrupole-Exactive Orbitrap mass spectrometry was used to analyze the extracted samples, which showed a broad scanning range of 215–1800 *m*/*z*. With MS-DIAL software and built-in LipidBlast database, we identified 38 lipids in positive, and 31 lipids in negative, ion mode, including lysophosphatidylcholine (lysoPC), phosphatidylcholine (PC), phosphatidylethanolamine (PE), phosphatidylglycerol (PG), etc. Then, the changes of lipids in BALF of mice with acute lung injury (ALI) induced by lipopolysaccharide (LPS) was investigated, which may contribute to further exploration of the pathogenesis of ALI.

## 1. Introduction

Lipidomics is an important branch of metabolomics, aiming at the comprehensive analysis of lipids in biological systems. It is a useful research tool that can be applied in lipid biochemistry, lipid-related clinical biomarker discovery, disease pathology study and disease diagnosis. For example, by comparing the changes in lipid metabolism networks under different physiological conditions, lipidomics assay can be used to identify the biomarkers in metabolic regulation, thus, revealing the mechanism of lipid action in various life activities. Lipidomics technology mainly includes the research of lipid extraction, isolation, analysis, identification and bioinformatics technology. Software is usually required to analyze sample similarities and differences to identify significant regulated molecules between samples or groups, which can further undergoe in-depth analysis of metabolic pathways, biomarker identification, and biological significance assessment [1,2,3,4,5].

Bronchoalveolar lavage fluid (BALF) is the fluid re-collected from bronchial and alveolar sterile saline is squirted into the lung segment or sub-segment level. The alveolar lavage fluid contains pulmonary epithelial secretions from the alveolar region including alveolar macrophages and other infiltrating inflammatory cells [6]. Pulmonary surfactant is a mixture of lipids and proteins secreted by alveolar type II epithelial cells. It contains approximately 90% lipids, mainly phosphatidylcholine, phosphatidylethanolamine, phosphatidylglycerol, and the like. Pulmonary surfactant lipids have functions such as maintaining lung stability, protecting lung tissue, preventing alveolar epithelial cell damage and fighting infection [7,8,9]. Scott Walmsley et al. described a prototypic small molecule databases for BALF-based metabolomics, including pulmonary surfactant lipids [10].

The metabolism of surfactant lipids plays an important role in maintaining the balance of alveolar microenvironment and lung immunity. Studies have shown that a variety of lipids may have metabolic abnormalities in human lung diseases [9]. For example, glycerol phospholipids, together with proteins, can form an immunologically active substance on the alveolar surface to participate in the body’s inflammatory reaction and lung infection [11]. Insufficient secretion of phosphatidylserine can lead to the lung diseases called Acute respiratory distress syndrome (ARDS) [12,13,14]. Therefore, the study of pulmonary surfactant lipids will provide new ideas for elucidating the pathogenesis of lung diseases as well as new guidance for the prevention and treatment of lung diseases.

Lipopolysaccharide (LPS), also known as endotoxin, is the main component of the outer wall of Gram-negative bacteria [15,16]. LPS infection is the first risk factor for lung injury and was first used to induce ALI in Animal model [17]. Tracheal instillation is frequently used to cause lesions on the lungs [15] with a more direct and obvious effect on the lung injury model [15]. A few studies indicated that ALI was often accompanied by a decrease in the concentration of pulmonary surfactant, reduction of pulmonary surfactant activity, and metabolic disorders of various lipids [9,18].

Currently, chromatography-mass spectrometry is the main advanced technology to analyze various complex lipid compounds [19,20,21]. In this paper, high-resolution mass spectrometry was used to establish a lipid analysis method for alveolar lavage fluid. Afterwards, the lipid changes in BALF samples from LPS induced ALI mice compared with controls were discovered.

## 2. Results

### 2.1. Evaluation of Mouse Model of Acute Lung Injury

**Figure 1:**
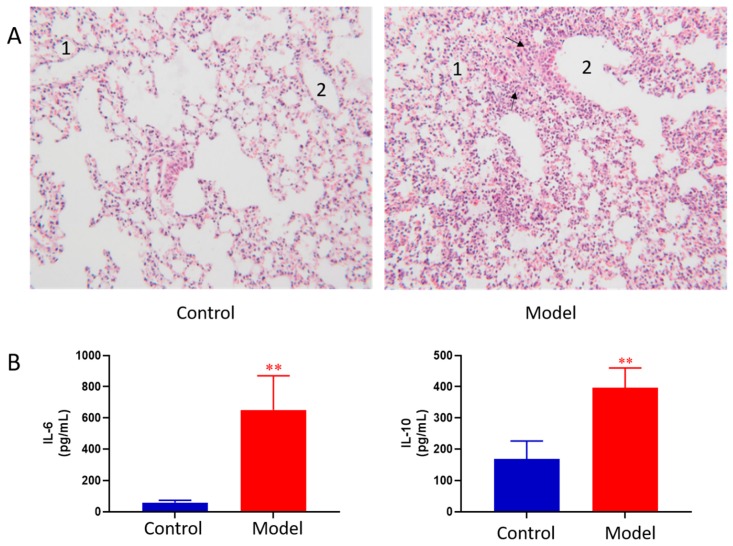
Pathological change of lung tissue of control and ALI model mice (HE × 100) 1, alveolar; 2, trachea; The black arrows indicate increased numbers of pulmonary macrophages and neutrophils (**A**), BALF cytokines of the control and ALI model mice. Values are expressed as mean ± SD. (*n* = 4), ** *p* < 0.01 vs. control mice (**B**).

The lung histopathology of mice in each group is shown in Figure 1A. The lungs of control group showed normal morphology with thin alveolar wall and no exudate in the bronchial cavity. The mucosal epithelium showed no degeneration and necrosis, and there was no inflammatory cell infiltration in the wall and surrounding tissues. In ALI model group, the lung morphology was abnormal. First, bronchial epithelial cells showed degeneration and necrosis. Second, the necrotic cells and exudate were seen in the cavity. Third, obvious inflammatory reaction and inflammatory cell infiltration were observed in pulmonary vascular and perivascular bronchi. Moreover, the granulocytes were obviously aggregated; at the same time, thickening of the alveolar wall and formation of early transparent membrane were also observed. Pathological results indicated that LPS induced ALI was successfully modeled.

BALF cytokines of the control and ALI model mice were determined. The results showed LPS induced ALI model led to concomitant increases in IL-6 and IL-10 as compared to the controls (Figure 1B). The results indicated that LPS induced ALI was successfully modeled.

### 2.2. Analysis of Lipid Components in Alveolar Lavage Fluid

**Figure 2:**
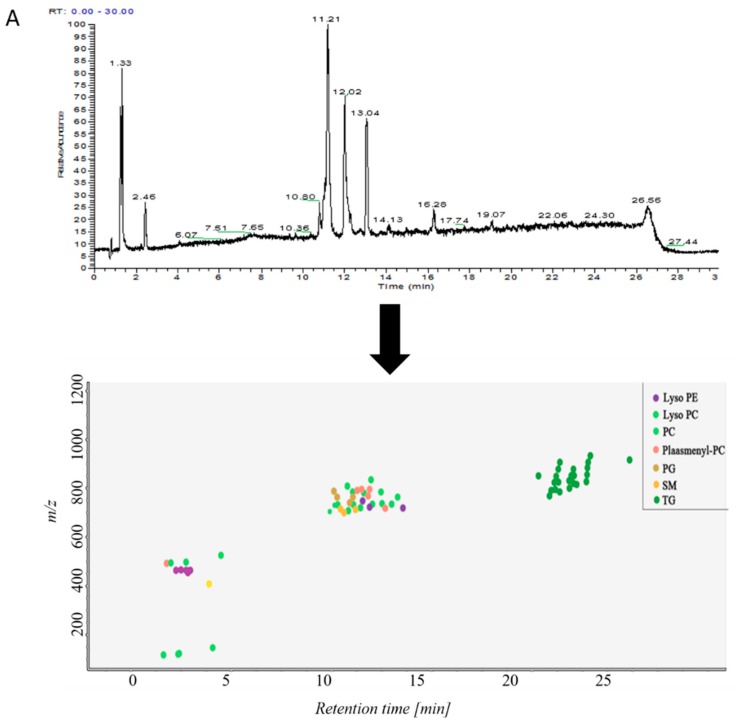

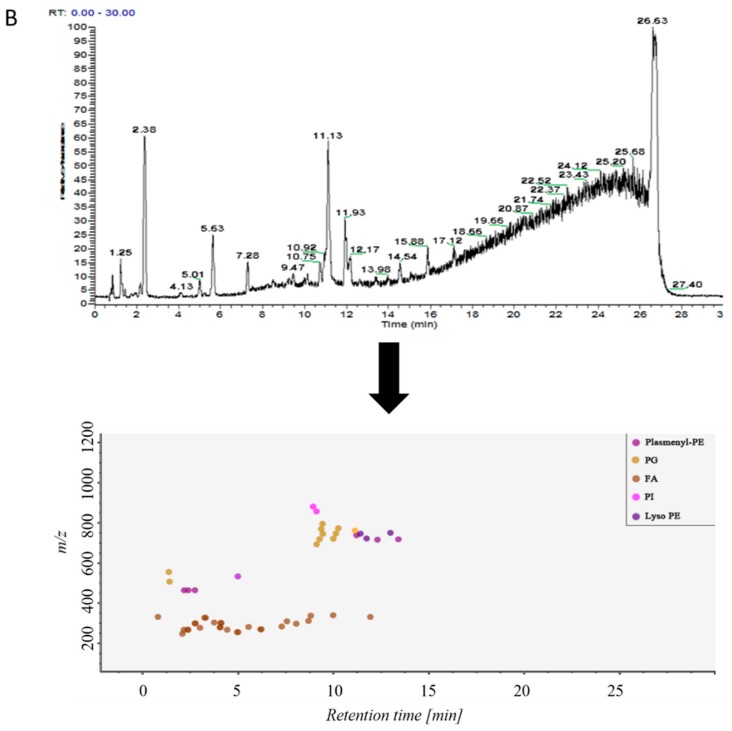
(**A**) TICs of lipids in BALF of mice and distribution of lipids in different retention times in positive ion mode in MS-DIAL software, (**B**) TICs of lipids in BALF of mice and distribution of lipids in different retention times in negative ion mode in MS-DIAL software.

Samples in control group and ALI model group were detected by hybrid quadrupole-exactive orbitrap mass spectrometry (UHPLC-Q-Exactive Orbitrap MS). The samples were randomized in both positive and negative ion mode. The raw data were analyzed using the MS-DIAL software and LipidBlast databases of over 200,000 MS/MS spectra. The total ion current map (TIC) and the distribution of lipid components at various time periods in positive ion mode are shown in Figure 2A. Figure 2B shows the TIC and distribution of lipids across the entire retention time in negative ion mode. Accurate mass and MS/MS matching with the public LipidBlast library were used for lipid annotation and identification.

The primary and secondary fragment ions were matched to the software’s built-in LipidBlast database (supplementary materials Figure S1). The figure showed the MS mass fragmentation map of selected lipids. The blue part above the 0-scale of the vertical axis was the data map of the LipidBlast database. The red part below the 0 scale was given by MS-DIAL. The data fragments of selected lipids were paired with the LipidBlast database. The fragments were matched with the LipidBlast database, indicating that the identification was accurate.

38 lipid molecules were covered in positive ion mode (Table S1): acylcarnitine; lysophosphatidylcholine (lysoPC); lysophosphatidylethanolamine (lysoPE), phosphatidylcholine (PC) Phosphatidylethanolamine (PE); phosphatidylglycerol (PG); plasmanoyl-PC (P-PC); sphingomyelin (SM) and triglycerides Lipids such as Triglyceride (TG).

31 lipid species were covered in negative ion mode (Table S2): fatty acid (FA); lysophosphatidylethanolamine (lysoPE); lysophosphatidylglycerol (lysoPG); phosphatidylethanolamine (PE); Phosphatidylglycerol (PG); phosphatidylinositol (PI); plasmenyl-phosphatidyl ethanolamine (plasmenyl-PE) and sphingomyelin (SM) lipids.

### 2.3. Liposomics of Alveolar Lavage Fluid in Mice with Acute Lung Injury

#### 2.3.1. UHPLC-Q-Exactive Orbitrap MS Method Validation

Three methods were used in this experiment to monitor the experimental operation error and investigate the stability of the instrument: (1) advanced 10-pin QC sample balance system before entering the experimental sample; (2) the peak height of the internal standards were monitored in all samples to calculate the RSD; (3) a blank solvent sample and QC sample were injected after each 10 experimental samples.

**Figure 3:**
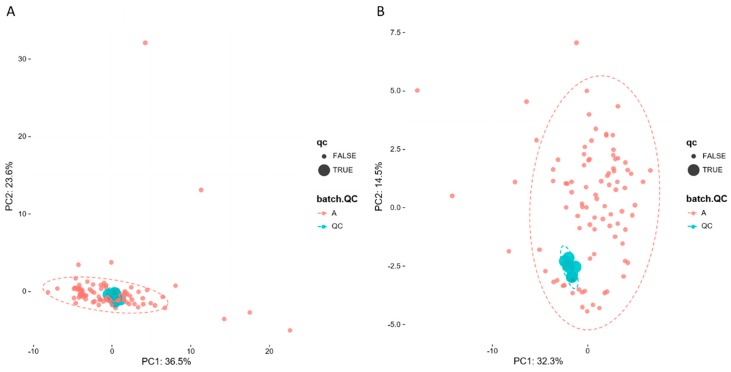
PCA of lipids in samples and QCs: (**A**) positive ion mod; (**B**) negative ion mod.

To evaluate system stability and reproducibility, PCA analysis was performed to process the data matrix of QC samples. As shown in Figure 3A,B, in PCA score plots of BALF samples, QC samples were clustered in both positive ionization and in negative ionization which indicated that the stability of the LC-MS system was good throughout the whole analysis. In addition, the relative standard deviations (RSDs) of the internal standards, such as lyso PE (17:1) and SM (17:0) in positive ion mode were 8.47% and 10.15%, and the RSDs lyso PE (17:1) and PE (17:0/17:0) were 12.22% and 9.91% in negative ion mode.

#### 2.3.2. Non-Targeted Lipidomics Metabolic Analysis

**Figure 4:**
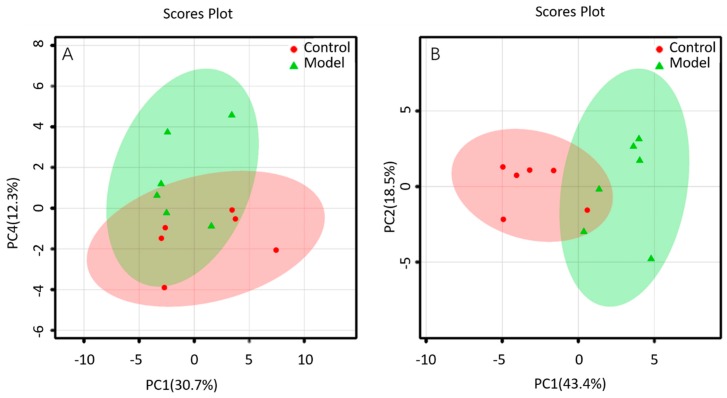
PCA of lipids in BALF of mice: (**A**) positive ion mod; (**B**) negative ion mod.

The lipid metabolites between the BALF of control group and that of ALI model group were compared in the positive and negative ion mode. The data sets of each group in the positive and negative ion modes were analyzed by PCA. Each dot in Figure 4 represents a sample. From the figure, preliminary PCA model of global lipid changes in BALF revealed consistent separation of ALI model group from normal controls in both positive ionization and in negative ionization (Figure 4A,B), suggesting that the BALF of control group and ALI model group do have metabolic differences.

#### 2.3.3. Analysis of Differential Lipid Metabolites

The U-test and FDR calibration were used to find the differential lipids in the BALF of control group and that of ALI model group. *p* < 0.05 and FDR < 0.2 were selected for the selection of differential lipids. In the positive ion mode, 11 differential lipids were obtained, e.g., lysoPC, PC, plasmenyl-PC, SM and TG (Table S3); 14 differential lipids were obtained in negative ion mode, namely FA, PE, PG, and plasmenyl-PE, et al. (Table S4).

**Figure 5:**
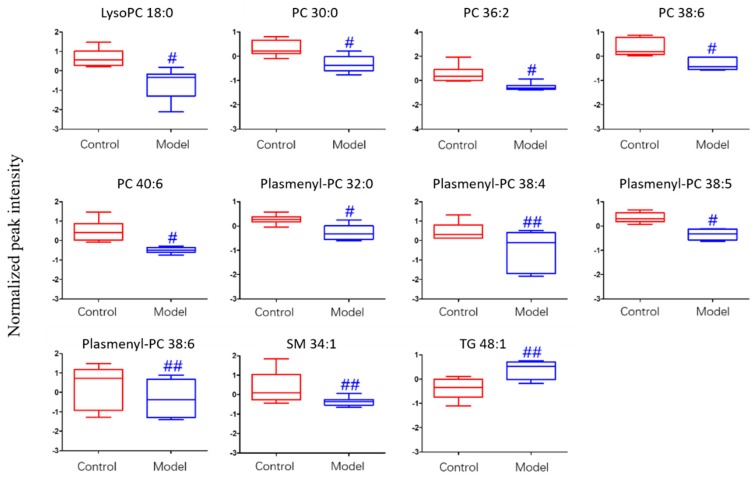
Box-whisker Plot of normalized peak intensity of differential lipids in BALF (positive ion mode) # *p* < 0.05, ## *p* < 0.01 vs. blank control mice.

**Figure 6:**
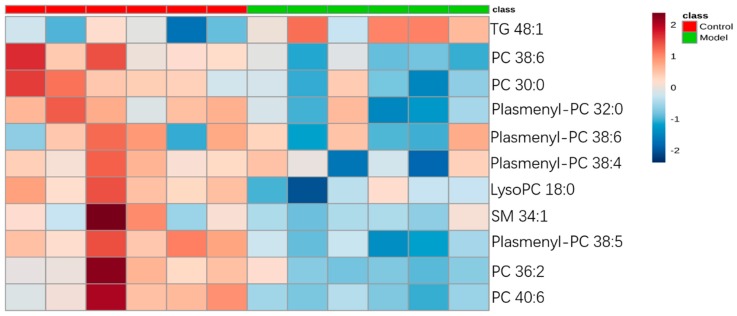
Heatmap of identified differential lipids in BALF (positive ion mode). Each square in the heatmap represents the corresponding intensity value of a phospholipid of samples in each group, red represents the increase in concentration and blue represents the decrease in concentration.

For the differential lipid analysis in positive ion mode, the relative species and variation trend of differential lipids in mice BALF are shown in Figure 5. The results show that there is a significant difference between control group and ALI model group (*p* < 0.05 or *p* < 0.01). The differential lipids in positive ion mode are clustered and the resulting heatmap is shown in Figure 6. Each square in the figure represents the corresponding intensity of each group of samples, red represents the increase in concentration and blue represents the decrease in concentration. The darker the color, the stronger the degree of increase/decrease in concentration of metabolites. It can be seen from the figure that control group is significantly different from ALI model group, and is divided into two bundles. Compared with control group, the TG of ALI model group is up-regulated, while the lysoPC, PC, plasmenyl-PC, and SM are all down-regulated. It is suggested that lipids such as lysoPC, PC, plasmenyl-PC, SM and TG in BALF of LPS-induced ALI model mice may have metabolic disorders.

**Figure 7:**
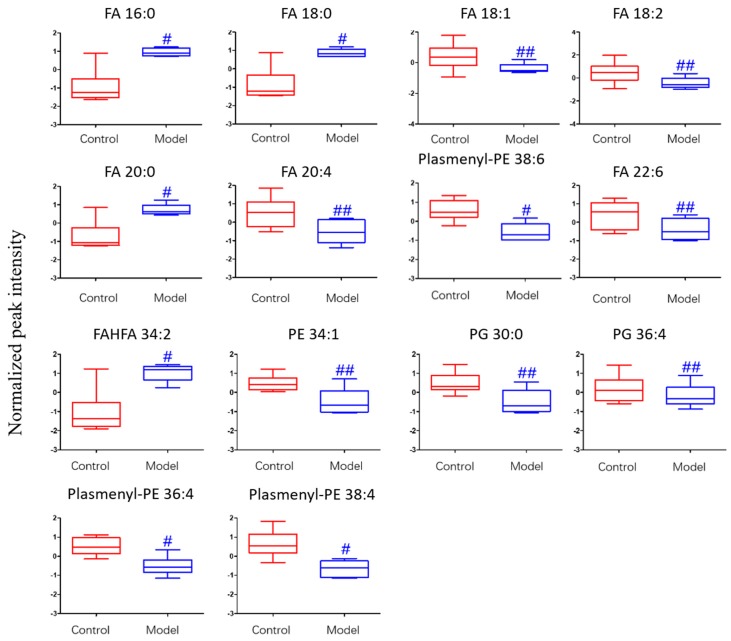
Box-whisker Plot of normalized peak intensity of differential lipids in BALF (negative ion mode) # *p* < 0.05, ## *p* < 0.01 vs. blank control mice.

**Figure 8:**
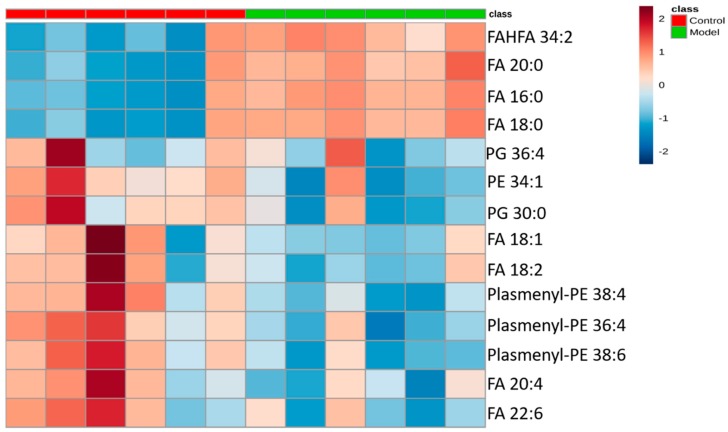
Heatmap of identified differential lipids in BALF (negative ion mode). Each square in the heatmap represents the corresponding intensity value of a phospholipid of samples in each group, red represents the increase in concentration and blue represents the decrease in concentration.

The differential lipids in negative ion mode were analyzed. The relative species and variation trend of differential lipids in mouse BALF are displayed in Figure 7. The results show that there is a significant difference between the blank group and the ALI model group (*p* < 0.05 or *p* < 0.01). The differential lipids in the negative ion mode are clustered and the resulting heat map is shown in Figure 8. Similar to positive ion mode, the lipids analyzed in negative ion mode further proved a significant difference between control group and ALI model group, and the heatmap is divided into two bundles. Compared with control group, some FAs (FA16:0, FA18:0, FA20:0) in ALI model group show an upward trend, while PE, PG, plasmenyl-PE and 4 other FAs show a downward trend. This result indicates that lipids such as FA, PE, PG, and plasmenyl-PE in BALF of LPS-induced ALI model mice may also have metabolic disorders.

## 3. Discussion

The relationship between lipid metabolism and lung diseases has attracted widespread attention. They are closely related to each other. Lipid metabolism imbalance can induce lung diseases, and lung diseases can also lead to lipid metabolism disorders [9,22]. It is possible to find new targets for the treatment of diseases if a deeper understanding of the relationship between lipid composition and lung disease is acquired [23]. Lipidomics is a rapidly evolving tool that explores the potential lipid biomarkers in diseases and the biological functions of lipids in various life activities by comparing the changes in lipid metabolism networks under different physiological conditions [24].

In the present study, we applied LC-MS based high-resolution lipidomics method to analyze potential disease relevant lipid alterations in BALF in ALI mice model. In our current study in total 69 lipid components were identified, with 38 lipid components in positive ion mode and 31 lipid components in negative ion mode such as lysoPC, PC, plasmenyl-PC, PE, plasmenyl-PE, PG, PI, etc. To further elucidate the relationship between these altered lipids and ALI disease, we screened out differential lipids. Compared to the blank control group, TG and some FAs were up regulated in BALF of the ALI model group. LysoPC, PC, plasmenyl—PC, PE, plasmenyl-PE, PG, SM and some FAs showed a downward trend in BALF of ALI model group.

We found that the most prominent manifestation in ALI model mice is the metabolic disorder of phospholipid lipids. The most abundant phospholipid component is PC. PC and surfactant proteins contribute to the surfactant activity. Its main function is to reduce surface tension, maintain alveolar stability, reduce respiratory resistance, and maintain ventilation efficiency [25]. The most abundant PC phospholipid species is PC (16:0/16:0), also known as DPPC. DPPC is associated with LPS-induced lung disease. DPPC can inhibit cytokine production induced by airway epithelial cells and LPS, potentially by incorporation into the plasma membrane and affecting cell membrane fluidity. In addition, intratracheal DPPC supplementation in mice attenuates lung inflammation induced by intravenous LPS [22,24]. Another major phospholipid component is PG, which plays a role in stabilizing the alveolar tension, and in regulating the innate immune response [26]. PG has a very good anti-inflammatory effect, which can inhibit the proinflammatory response of macrophages to LPS by interfering with the LBP-CD14-TLR4 complex. LysoPC is also an important constituent of surface active substances with important regulatory role in inflammatory reactions. It may regulate the inflammatory response caused by LPS [27]. Consistent with previous review article [22], PC and PG are metabolically disordered and down-regulate. LPS inhibited the synthesis and secretion of phospholipids in our study. Another prominent manifestation of ALI model mice was the increase of part the fatty acid species. The same decreasing trend of PC and lysoPC indicated possible metabolic conversion of those lipids into their corresponding free fatty acids, which may further trigger a series of acidosis [27].

TG is the most abundant lipid in the human body. In addition to providing energy, its metabolic disorder is closely related to chronic inflammatory reactions in the lungs [28]. It was slightly strange, in our study, LPS-induced ALI model did not change significantly most of TG except for TG 48:1.

SM is called “bioactive lipids”. It is a type of substance synthesized in the inflammatory reaction. The abnormality of some SM leads to a series of sputum effects, which cause allergic and inflammatory reactions. SM coordinates the delicate balance between host and the microbe. The microbe utilizes the sphingolipids of the host cell to enhance pathogenicity, and the host body can defend against microbial attack with the participation of SM [28].

In addition, we have also identified plasmalogen lipids. These lipids can prevent lung disease due to their antioxidant effects, as the lung is a direct target of reactive oxidant species [24]. In this study, we found that the concentration of plasmalogen lipids decreased. It suggests that plasmalogen lipids may play a key role of normal lung physiology.

**Figure 9:**
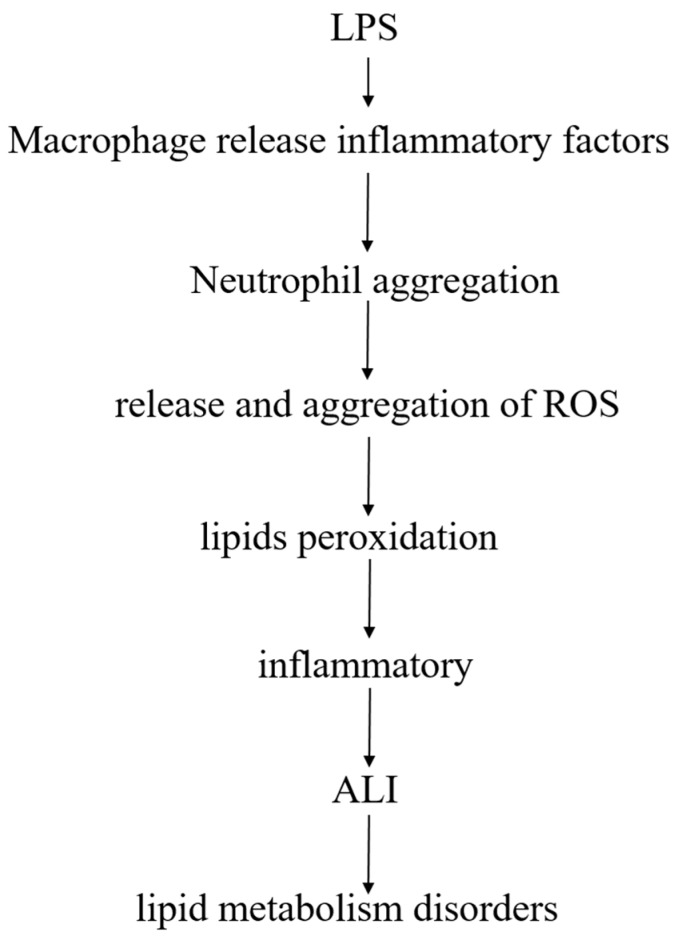
The relationship between lipids and LPS-induced ALI.

Lipids are closely linked to LPS-induced ALI model, after LPS enters the body, pulmonary macrophages release inflammatory factors after stimulation, and promote polymorphonuclear leukocytes (PMN, mainly neutrophils) to accumulate in the lungs. After activation of PMN, release of reactive oxygen species (ROS), excess ROS may cause lipids peroxidation, produce an inflammatory response, and damage lung tissue, leading to ALI, accompanied by a phenomenon of lipid metabolism disorder (Figure 9).

## 4. Materials and Methods

### 4.1. Instruments and Chemicals

Revco UXF ultra-low temperature refrigerator (Thermo Fisher Scientific, San Jose, CA, USA); CPA225D type one hundred thousandth electronic balance (Sartorius, Göttingen, Germany); ALLegra64R high speed refrigerated centrifuge (Beckman, Pasadena, CA, USA); THZ-C constant temperature oscillator (Taicang Qianle Experimental Equipment Co., Ltd., Taicang, JangSu, China); KQ-500B ultrasonic cleaner (Kunshan Ultrasonic Instrument Co., Ltd., Kunshan, JangSu, China); Savant SPD1010 vacuum centrifugal concentrator (Thermo Fisher Scientific, San Jose, CA, USA); U3000 efficient Liquid Chromatograph (Dionex, Santa Clara, CA, USA); Q-Exactive Quadrupole—Electrostatic Field Orbitrap High Resolution Mass Spectrometer (Thermo Fisher Scientific, San Jose, CA, USA); Xcalibur 2.1 SP1 Data Processing System (Thermo Fisher Scientific, San Jose, CA, USA).

Lipopolysaccharide (Sigma, Santa Clara, CA, USA); phosphate buffered saline (PBS): sodium chloride 8.0 g, potassium chloride 0.2 g, disodium hydrogen phosphate 1.56 g, potassium dihydrogen phosphate 0.1 g, dissolved in ultrapure water and brought to volume 1000 mL, sterilized in a high-pressure steam sterilizer, cooled at room temperature; methyl tert-butyl ether (MTBE) purchased from ROE, USA; internal standard: lyso PE (17:1) (batch number: LM171LPE-11), SM (17:0) (batch number: 170SM-13), PE (17:0/17:0) (batch number: LM170PE-19) purchased from Avanti Polar Lipids Company; glacial acetic acid (batch number: 15080553613, purity: 99.5%) was purchased from Nanjing Chemical Reagent Co., Ltd.; isopropanol, ammonium formate and ammonium acetate were both 99.8% mass spectrometry pure, purchased from ROE Company of the United States; methanol and acetonitrile were 99.8% mass spectrometry, purchased from Merck, Germany.

### 4.2. Animals

SPF C57BL/6 male mice, 12, weighing 18–22 g, 6–8 weeks old, purchased from Nanjing Qinglongshan animal farm, animal certificate number: SCXK-Su-2016-0003. The mice were housed in an environment with a room temperature of 24–26 °C, a relative humidity of 55%, and a 12-h light-dark cycle. The mice were kept in separate cages and had free access to water and given food to acclimatize before initiating experiments. The animal experiments in this study were approved and adhered to the Institutional guidelines for Animal Care and Use Committee of Nanjing University of Chinese Medicine. The project identification code of the ethical statement is 201809A029.

Three days after adaptive feeding, the mice were randomly divided into two groups: ALI model group (LPS tracheal instillation group, *n* = 6) and control group (saline tracheal instillation group, *n* = 6). All mice were intraperitoneally injected (10 mL·kg^−1^) with a 4% chloral hydrate solution for anesthesia. For ALI model group, the mice were instilled with 50 μL of LPS solution at a concentration of 1.5 mg·mL^−1^. (Dose was 3 mg·kg^−1^); the mice in the blank control group were instilled with the same volume of normal saline, and the mice were sacrificed 6 h later to collect the samples [29].

### 4.3. Sample Collection

Mice were euthanized, an incision was made from the middle of the neck, the neck skin muscles were separated, the trachea was exposed, and the catheter was inserted into the trachea. PBS (3 × 500 μL) was instilled into the airways and a consistent volume of BALF (1400–1450 μL) was recovered from each animal [24]. The lavage was centrifuged at 1500 rpm at 4 °C, and the BALF was stored at −80 °C for further analyses. The lobules of lung were collected for histopathological examination and the others was stored at −80 °C.

### 4.4. Histopathology

The lung tissues from each mouse were fixed in 4% neutral buffered paraformaldehyde for 12 h dehydration, then embedded in paraffin wax to slice into 3 μm thick sections and stained by hematoxylin and eosin (H&E) staining. Tissue lesions and inflammatory cell infiltrations were examined using a microscope.

### 4.5. Measurements of Inflammatory Mediators

The cytokine levels of IL-6 and IL-10 were evaluated by ELISA kits in accordance.

### 4.6. Sample Preparation

We used a sample preparation strategy for covering different classes of lipids based on the liquid–liquid MTBE extraction to analysis lipidomic [30,31,32].

Briefly, BALF (20 μL) was first added to a 1.5 mL centrifuge tube followed by 225 μL ice methanol solution with internal standard (lysoPE (17:1), SM (17:0) for positive ion mode and PE (17:0/17:0) for negative ion mode, internal standard concentration is about 5 μg/mL), the mixture was vortexed for 10 s. Next, 750 μL MTBE was added, and the mixture were shaken for 10 min at 4 °C. After adding 188 μL of deionized water, the samples were vortexed for 10 s and then centrifuged at 14,000 rpm at 4 °C. The upper (organic) phase, mainly lipids were transferred to fresh tubes and dried in a vacuum centrifuge. Finally, the upper phase lipids were reconstituted with 110 μL methanol: toluene (9:1) for LC-MS analysis.

### 4.7. Untargeted Lipidomic Analysis

To detect lipids, 2 µL aliquots of sample solution was injected onto a reversed phase Waters Acquity UPLC CSH C18 (100 mm × 2.1 mm, 1.7 μm) maintained at 60 °C by gradient elution. Mobile phase A was water: ACN (6:4), and mobile phase B was isopropanol: ACN (9:1), both containing 10 mM ammonium formate and 0.1% formic acid. The flow rate was 0.3 mL/min, with the elution gradient as follows: 0–4.0 min, 15% B; 4.0–5.0 min, 15–48% B; 5.0–22.0 min, 48%–82% B; 22.0–23.0 min, 82–99% B; 23.0–24.0 min, 99% B; 24.0–24.2 min, 99%–15% B; 24.2–30.0 min, 15% B [24].

The source and ion transfer parameters applied were as followed: spray voltage 3.5 kV (positive) and 3.0 kV (negative). For both ionization modes, the sheath gas, aux gas, the capillary temperature and the heater temperature were maintained at 35, 15 (arbitrary units), 325 °C and 300 °C, respectively [24].

### 4.8. Data Processing

After lipid annotation, a small-scale database was set up with lipid name, retention time (RT) and accurate mass to charge ratios (*m*/*z*). Raw data files acquired from Xcalibur 2.2 software (Thermo Scientific) were converted to ABF format using the ABF converter (accessible at: http://www.reifycs.com/AbfConverter). For data processing, MS-DIAL (v. 2.78) software program was used. In this study, only a lipid feature defined as an *m*/*z*—RT pair can be aligned for an identical lipid. The resulting output data table of high-quality time-aligned examined lipids, with their corresponding RT, m/z and peak area acquired for each sample, was subjected to further statistical analysis [24].

### 4.9. Statistical Analysis

Multivariate analysis was performed using MetaboAnalyst 4.0 (http://www.metaboanalyst.ca 19). The loess normalization of the data matrix based on the R language was used [33,34]. Principal component analysis and CA was performed, and model evaluation with permutation strategy was carried out. The differential lipids were selected by U-test and FDR calibration with *p* value < 0.05 and FDR < 0.2.

## 5. Conclusions

In summary, we established a lipidomics analysis method for mice BALF using UHPLC-Q-Exactive Orbitrap MS high-resolution mass spectrometry. With MS-DIAL software and LipidBlast database, we identified 38 and 31 different lipids in positive and negative ion modes, respectively. The main components are phospholipids, sphingomyelin, and fatty acids, etc. Through the identification and analysis of lipids, it is speculated that LPS-induced ALI may lead to metabolic disorders of lipids in the body, especially pulmonary surfactant lipids, indicating that lipids play an important role in the pathogenesis of ALI. However, our study also has certain limitations. It is a non-target experiment, so the relative quantification of the substance is taken, and the substance is not absolutely quantified. In the future, we think it would be necessary to analyze the inflammation-related lipids in biological samples to explore their roles in cellular functioning and pathophysiological events.

## Supplementary Materials

The following are available online at https://www.mdpi.com/2218-1989/9/4/80/s1, Figure S1. Selected lipids with matching fragmentation patterns to the LipidBlast library with MS-DIAL software.
