# Supplementary material for: Surfactant Lipidomics of Alveolar Lavage Fluid in Mice Based on Ultra-High-Performance Liquid Chromatography Coupled to Hybrid Quadrupole-Exactive Orbitrap Mass Spectrometry
Source: Metabolites. 2019 Apr 25;9(4):80. doi: 10.3390/metabo9040080 (PMC6523637; doi:10.3390/metabo9040080)
Supplement: Supplementary file 1 [file metabolites-09-00080-s001.pdf]

SUPPORTING INFORMATION

Surfactant Lipidomics of Alveolar Lavage Fluid in Mice Based on Ultra-High-Performance Liquid Chromatography Coupled to Hybrid Quadrupole-Exactive Orbitrap Mass Spectrometry

Rui Yang [1,2,3] , Ying Zhang[4], Wenjuan Qian[1,2] , Linxiu Peng [2,3], Lili Lin [2,3], Jia Xu [2], Tong Xie[2,3] , Jianjian Ji[3], Xiuqin Zhan[1*] and Jinjun Shan[1,2,3*]

[1] Jiangsu Key Laboratory of Pediatric Respiratory Disease, School of Medicine and Life Sciences, Nanjing University of Chinese Medicine, Nanjing 210023, China
[2] Medical Metabolomics Center, Nanjing University of Chinese Medicine, Nanjing 210023, China
[3] Institute of Pediatrics, Nanjing University of Chinese Medicine, Nanjing 210023, China
[4] Genome Center of UC Davis，NIH West Coast Metabolomics Center，Davis 95616，USA
* Correspondence: jshan@njucm.edu.cn; Tel.: 86-25-85811329; xqzhan14@sina.com

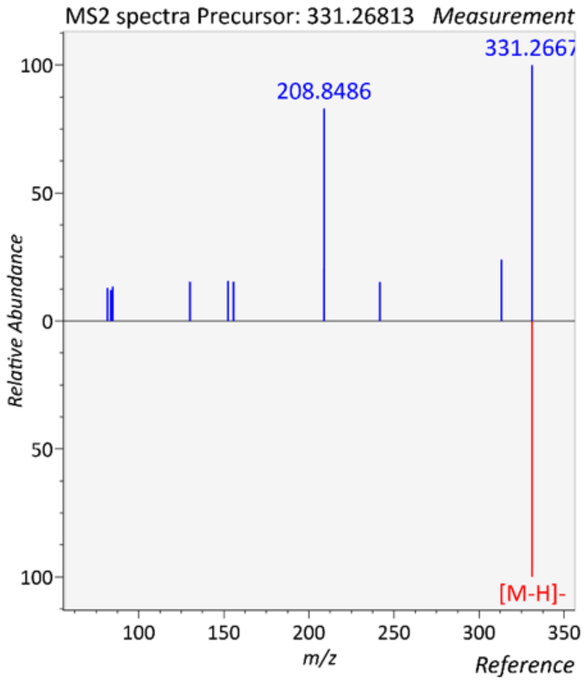

FA 22:4

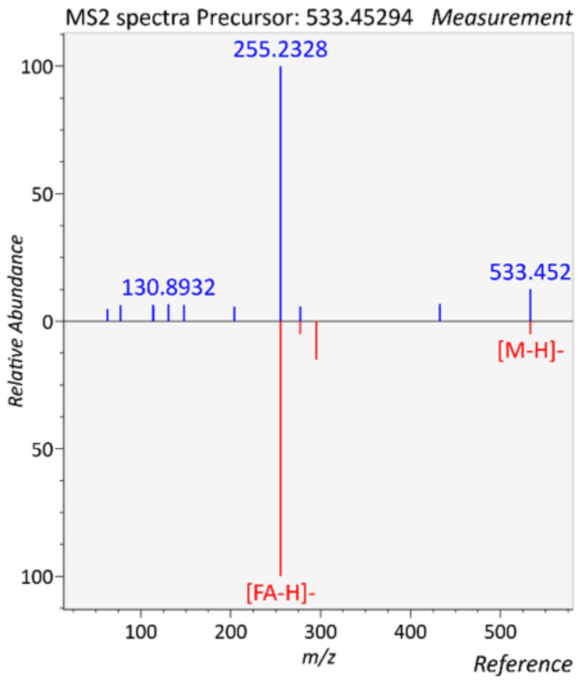

FAHFA 34:2

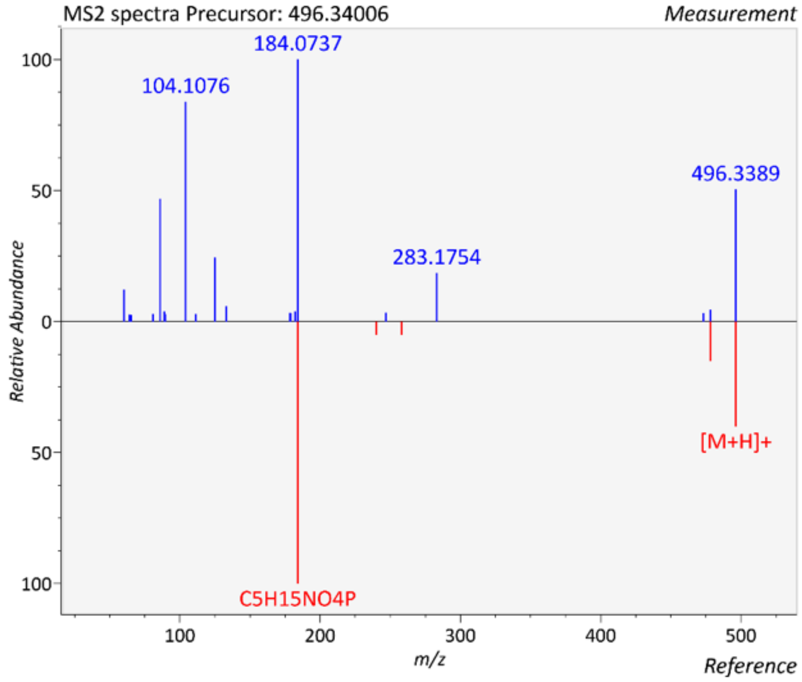

LPC 16:0

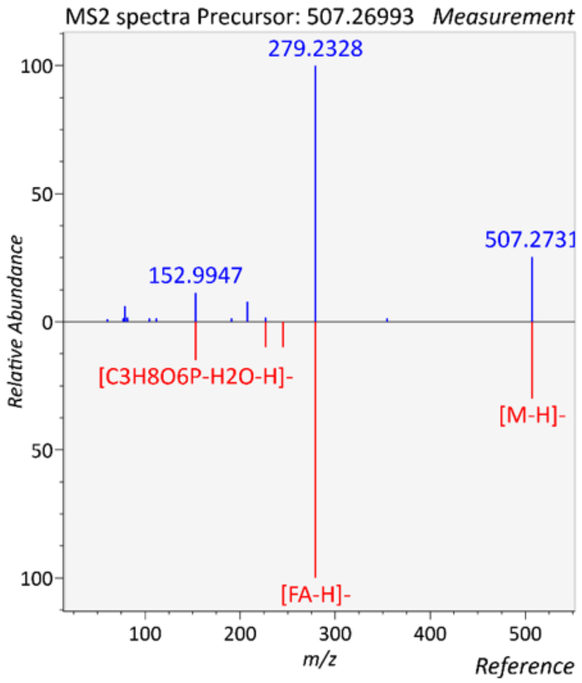

LPG18:2

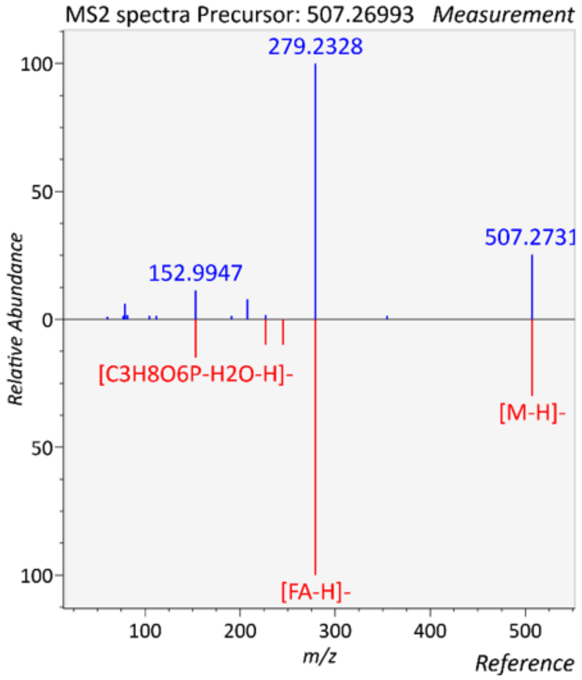

PC 36:5

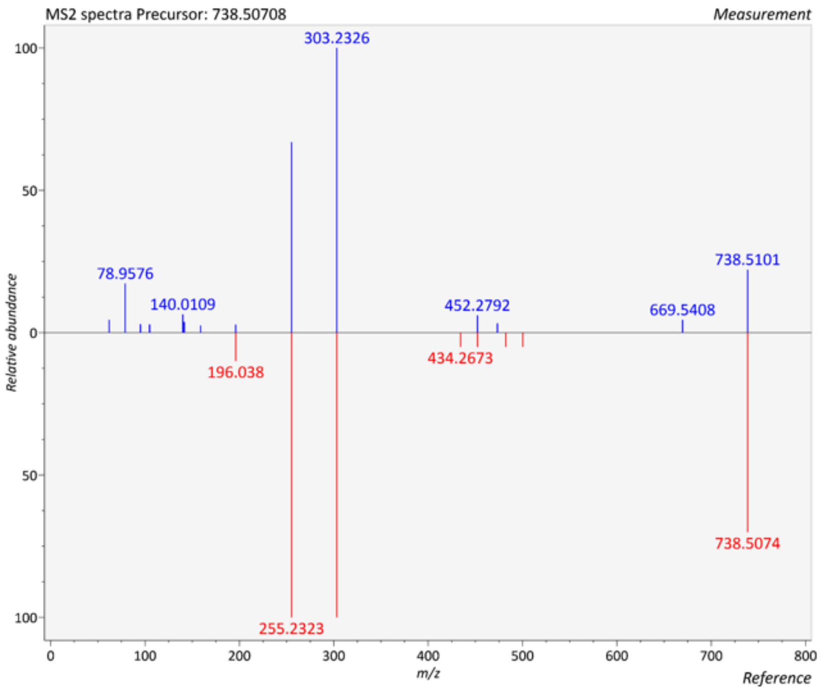
MS2 spectra Precursor: 738.50708
Measurement
303.2326
78.9576
140.0109
452.2792
669.5408
738.5101
196.038
434.2673
738.5074
255.2323
Relative abundance
m/z
Reference

PE 36:4

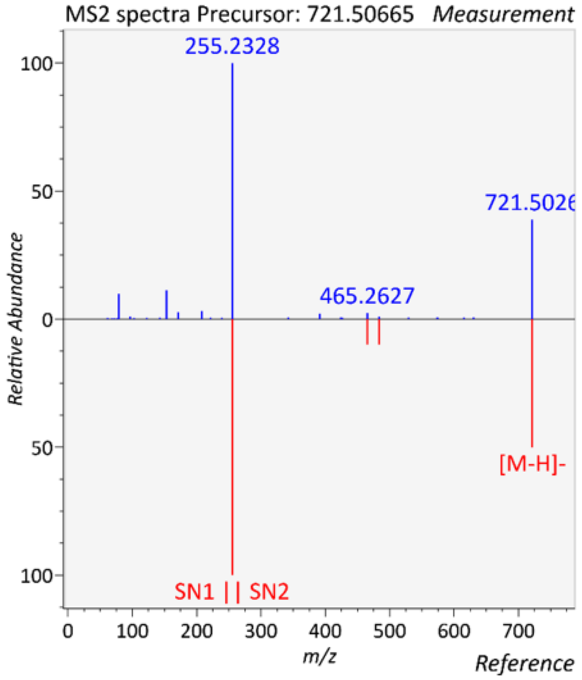
MS2 spectra Precursor: 721.50665
Measurement
255.2328
721.5026
465.2627
[M-H]-
SN1 | | SN2
Relative Abundance
m/z
Reference

PG 32:0

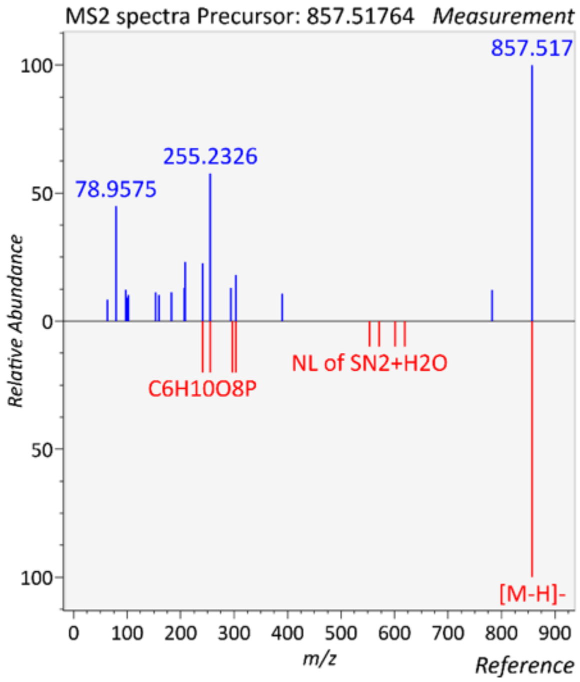
MS2 spectra Precursor: 857.51764 Measurement
857.517
255.2326
78.9575
C6H10O8P
NL of SN2+H2O
[M-H]-
Relative Abundance
m/z
Reference

PI 36:4

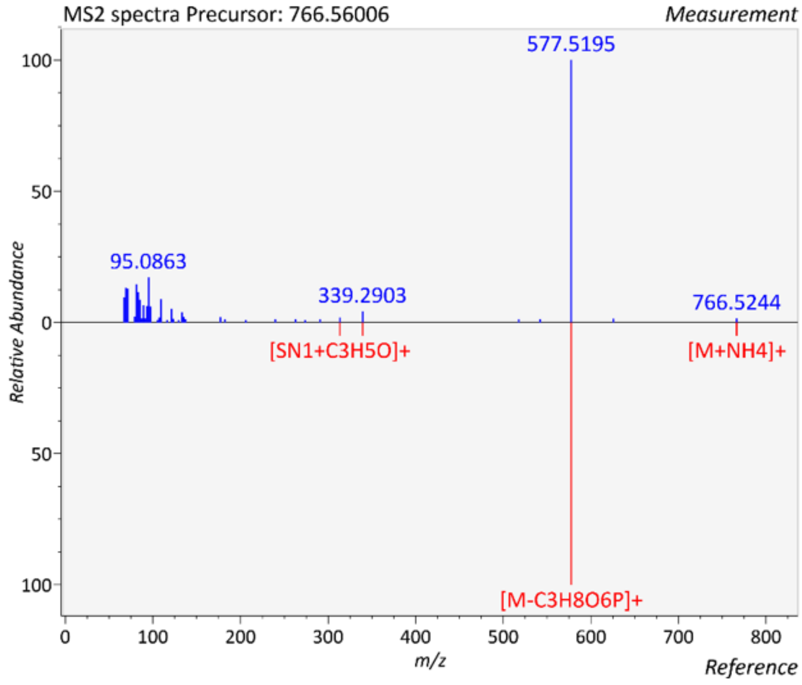
MS2 spectra Precursor: 766.56006
Measurement
577.5195
95.0863
339.2903
766.5244
[SN1+C3H5O]+
[M+NH4]+
[M-C3H8O6P]+
Relative Abundance
m/z
Reference

PG 34:1

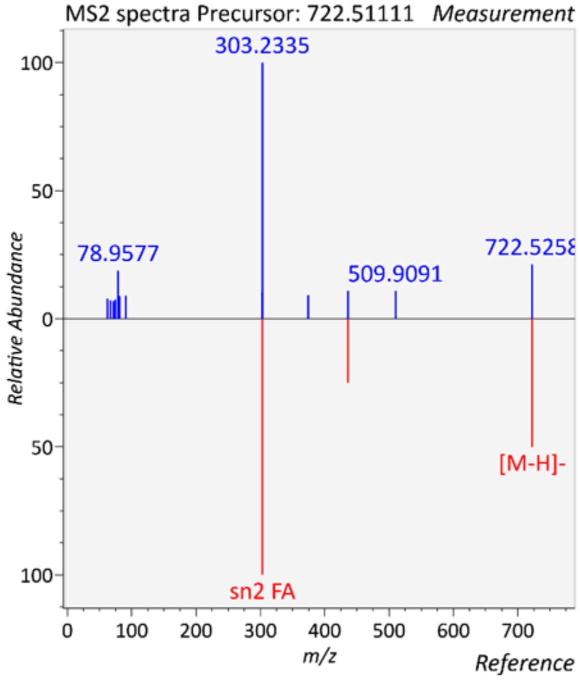

P-PE 36:4

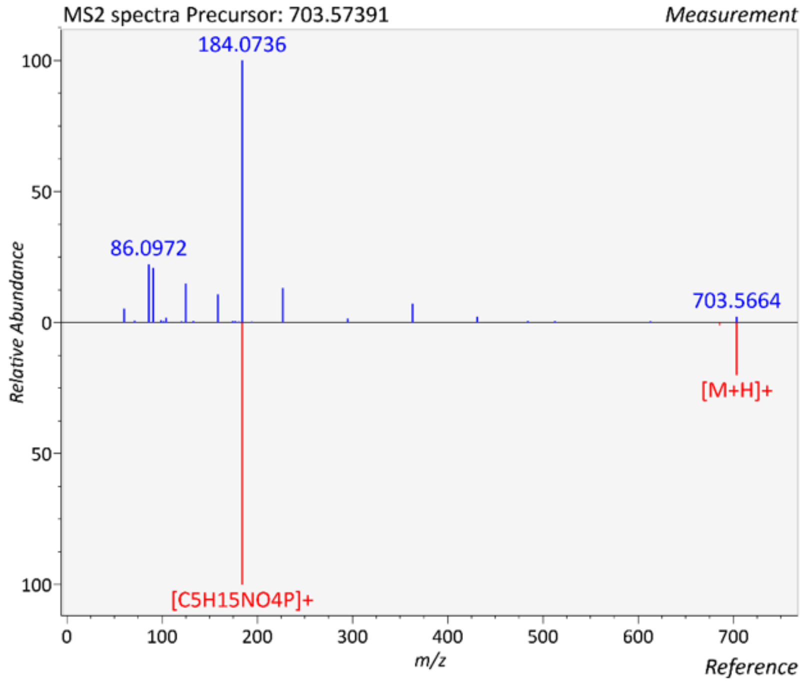

SM 34:1

Figure S1. Selected lipids with matching fragmentation patterns to the LipidBlast library with MS-DIAL software

Table S1. Lipids in BALF (positive ion mode)

Alignment ID	Metabolite name	Average Mz	Average Rt(min)	Adduct ion name	Delta ppm	Formula
6474	LysoPC 16:0	496.3398	2.77	[M+H]+	1.640	C24H50NO7P
6427	LysoPC 16:1	494.3263	1.98	[M+H]+	1.141	C24H48NO7P
7415	LysoPC 18:0	524.3699	4.49	[M+H]+	-0.641	C26H54NO7P
13397	PC 30:0	706.5400	10.81	[M+H]+	0.805	C38H76NO8P
14592	PC 32:0	734.5719	12.00	[M+H]+	0.529	C40H80NO8P
14527	PC 32:1	732.5554	11.06	[M+H]+	0.572	C40H78NO8P
14417	PC 32:2	730.5374	10.14	[M+H]+	0.861	C40H76NO8P
16475	PC 36:2	786.6042	12.47	[M+H]+	0.430	C44H84NO8P
16415	PC 36:3	784.5880	11.59	[M+H]+	1.094	C44H82NO8P
16346	PC 36:4	782.5710	11.08	[M+H]+	-0.283	C44H80NO8P
17235	PC 38:6	806.5706	10.80	[M+H]+	0.792	C46H80NO8P
18282	PC 40:6	834.5992	11.97	[M+H]+	4.432	C48H84NO8P
15719	PG 32:0	740.5450	10.90	[M+NH4]+	-1.932	C38H75O10P
15642	PG 34:1	766.5604	11.06	[M+NH4]+	4.148	C40H77O10P
16559	PG 34:2	764.5449	10.27	[M+NH4]+	0.758	C40H75O10P
13801	PG 36:4	788.5417	10.13	[M+NH4]+	-1.814	C42H75O10P
16800	Plasmenyl-PC 32:0	718.5762	12.63	[M+H]+	1.368	C40H80NO7P
16723	Plasmenyl-PC 38:4	794.6082	11.89	[M+H]+	-0.059	C46H84NO7P
14034	Plasmenyl-PC 38:5	792.5905	11.48	[M+H]+	0.282	C46H82NO7P
15064	Plasmenyl-PE 36:4	724.5272	11.88	[M+H]+	0.198	C41H74NO7P
13285	Plasmenyl-PE 38:6	748.5282	11.56	[M+H]+	1.902	C43H74NO7P
15996	SM 34:1	703.5757	10.61	[M+H]+	3.267	C39H79N2O6P
16355	TG 44:0	773.6679	20.87	[M+Na]+	1.976	C47H90O6
16883	TG 45:0	782.7265	21.38	[M+NH4]+	0.605	C48H92O6
16806	TG 46:0	796.7429	21.84	[M+NH4]+	0.344	C49H94O6
18054	TG 46:1	794.7216	21.09	[M+NH4]+	1.527	C49H92O6
17991	TG 48:0	829.7263	22.70	[M+Na]+	1.432	C51H98O6

17943	TG 48:1	827.7124	22.00	[M+Na]+	0.940	C51H96O6
17942	TG 48:2	825.6949	21.26	[M+Na]+	1.342	C51H94O6
19075	TG 48:2	825.6947	21.14	[M+Na]+	0.834	C51H94O6
18795	TG 50:1	855.7434	22.74	[M+Na]+	0.781	C53H100O6
18717	TG 50:3	846.7567	21.18	[M+NH4]+	2.449	C53H96O6
19970	TG 52:2	881.7590	22.77	[M+Na]+	-1.204	C55H102O6
19687	TG 52:3	874.7850	22.05	[M+NH4]+	0.016	C55H100O6
21070	TG 54:0	913.8254	24.85	[M+Na]+	2.733	C57H110O6
20843	TG 54:3	907.7659	22.81	[M+Na]+	-7.383	C57H104O6
20708	TG 54:5	903.7408	21.36	[M+Na]+	0.131	C57H100O6
21597	TG 56:4	928.8336	22.89	[M+NH4]+	-1.643	C59H106O6

Table S2. Lipids in BALF (negative ion mode)

Alignment ID	Metabolite name	Average Mz	Average Rt(min)	Adduct ion name	Delta ppm	Formula
2671	FA 16:0	255.2322	4.96	[M-H]-	0.623	C16H32O2
2083	FA 16:4	247.1706	2.09	[M-H]-	1.074	C16H24O2
3642	FA 18:0	283.2637	7.29	[M-H]-	0.543	C18H36O2
3537	FA 18:1	281.2481	5.55	[M-H]-	1.153	C18H34O2
3464	FA 18:2	279.2326	4.05	[M-H]-	1.173	C18H32O2
3422	FA 18:3	277.2176	3.01	[M-H]-	0.863	C18H30O2
4666	FA 20:0	311.2953	8.69	[M-H]-	0.773	C20H40O2
4622	FA 20:1	309.2794	7.56	[M-H]-	0.273	C20H38O2
4432	FA 20:4	303.2328	3.75	[M-H]-	0.563	C20H32O2
4385	FA 20:5	301.2169	4.09	[M-H]-	0.683	C20H30O2
4229	FA 20:6	299.2016	2.74	[M-H]-	0.893	C20H28O2
5477	FA 22:0	339.3261	9.98	[M-H]-	0.783	C22H44O2
5134	FA 22:6	327.2335	3.25	[M-H]-	1.173	C22H32O2
12271	FAHFA 34:2	533.4561	4.99	[M-H]-	-1.727	C34H62O4

11416	LysoPG 18:2	507.2730	1.41	[M-H]-	1.054	C24H45O9P
12907	LysoPG 22:6	555.2728	1.37	[M-H]-	-0.876	C28H45O9P
21445	PE 34:1	716.5248	12.31	[M-H]-	2.119	C39H76NO8P
24305	PE 36:4	738.5108	11.21	[M-H]-	1.839	C41H74NO8P
20207	PG 30:0	693.4739	9.12	[M-H]-	1.139	C36H71O10P
21729	PG 32:0	721.5031	10.00	[M-H]-	1.029	C38H75O10P
21602	PG 32:1	719.4894	9.27	[M-H]-	0.689	C38H73O10P
24913	PG 34:1	747.5191	10.13	[M-H]-	1.069	C40H77O10P
24721	PG 34:2	745.5032	9.44	[M-H]-	0.909	C40H75O10P
26511	PG 36:2	773.5331	10.25	[M-H]-	1.959	C42H79O10P
26283	PG 36:4	769.4990	9.36	[M-H]-	1.759	C42H75O10P
28284	PG 38:5	795.5177	9.43	[M-H]-	0.759	C44H77O10P
34558	PI 36:4	857.5194	9.11	[M-H]-	3.415	C45H79O13P
37365	PI 38:6	881.5167	8.94	[M-H]-	4.275	C47H79O13P
21740	Plasmenyl-PE 36:4	722.5110	11.74	[M-H]-	2.064	C41H74NO7P
25097	Plasmenyl-PE 38:4	750.5432	12.99	[M-H]-	0.603	C43H78NO7P
24801	Plasmenyl-PE 38:6	746.5122	11.41	[M-H]-	1.574	C43H74NO7P

Table S3. Differential lipids in BALF (positive ion mode)

Significant Metabolites	m/z	Rt(min)	Adduct ion	P value	FDR	Fold Change
LysoPC 18:0	524.3699	4.49	[M+H]+	<0.01	0.05	0.56
PC 30:0	706.5400	10.81	[M+H]+	0.01	0.06	0.82
PC 36:2	786.6042	12.47	[M+H]+	<0.01	0.05	0.60
PC 38:6	806.5706	10.80	[M+H]+	<0.01	0.05	0.82
PC 40:6	834.5992	11.97	[M+H]+	0.01	0.05	0.68
Plasmenyl-PC 32:0	718.5762	12.63	[M+H]+	0.01	0.06	0.86
Plasmenyl-PC 38:4	794.6082	11.89	[M+H]+	0.03	0.18	0.77
Plasmenyl-PC 38:5	792.5905	11.48	[M+H]+	<0.0001	0.01	0.83
Plasmenyl-PC 38:6	790.576	11.26	[M+H]+	0.01	0.09	0.51
SM 34:1	703.5757	10.26	[M+H]+	0.04	0.18	0.80
TG 48:1	827.7124	22.00	[M+Na]+	0.03	0.18	1.41

Table S4. Differential lipids in BALF (negative ion mode)

Significant Metabolites	m/z	Rt(min)	Adduct ion	P value	FDR	Fold Change
FA 16:0	255.2322	4.96	[M-H]-	<0.01	0.01	5.76
FA 18:0	283.2637	7.29	[M-H]-	<0.01	0.01	4.73
FA 18:1	281.2481	5.55	[M-H]-	0.04	0.11	0.64
FA 18:2	279.2326	4.05	[M-H]-	0.03	0.09	0.53
FA 20:0	311.2953	8.69	[M-H]-	<0.01	0.01	3.05
FA 20:4	303.2328	3.75	[M-H]-	0.02	0.06	0.52
FA 22:6	327.2335	3.25	[M-H]-	0.03	0.09	0.57
FAHFA 34:2	533.4561	4.99	[M-H]-	<0.01	0.01	11.42
PE 34:1	716.5248	12.31	[M-H]-	0.04	0.11	0.58
PG 30:0	693.4739	9.12	[M-H]-	0.03	0.09	0.59
PG 36:4	769.4990	9.36	[M-H]-	0.05	0.11	0.81
Plasmenyl-PE 36:4	722.5110	11.74	[M-H]-	0.01	0.02	0.59
Plasmenyl-PE 38:4	750.5432	12.99	[M-H]-	<0.01	0.01	0.49
Plasmenyl-PE 38:6	746.5122	11.41	[M-H]-	<0.01	0.01	0.54
